# Effects of High-Canolol Phenolic Extracts on Fragrant Rapeseed Oil Quality and Flavor Compounds during Frying

**DOI:** 10.3390/foods12040827

**Published:** 2023-02-15

**Authors:** Lili Cao, Pengpeng Jia, Haotian Liu, Shengmei Kang, Shaotong Jiang, Min Pang

**Affiliations:** 1 School of Food and Biological Engineering, Hefei University of Technology, Hefei 230009, China; 2 Key Laboratory for Agricultural Products Processing of Anhui Province, Hefei 230009, China

**Keywords:** canolol, fragrant rapeseed oil, frying process, flavor compounds

## Abstract

Fragrant rapeseed oil (FRO) is a frying oil widely loved by consumers, but its quality deteriorates with increasing frying time. In this study, the effect of high-canolol phenolic extracts (HCP) on the physicochemical properties and flavor of FRO during frying was investigated. During frying, HCP significantly inhibited the increase in peroxide, acid, p-anisidine, and carbonyl values, as well as total polar compounds and degradation of unsaturated fatty acids. A total of 16 volatile flavor compounds that significantly contributed to the overall flavor of FRO were identified. HCP was effective in reducing the generation of off-flavors (hexanoic acid, nonanoic acid, etc.) and increased the level of pleasant deep-fried flavors (such as (E,E)-2,4-decadienal). Therefore, the application of HCP has a positive effect on protecting the quality and prolonging the usability of FRO.

## 1. Introduction

As one of the world’s top three vegetable oils, rapeseed oil is popular among consumers because of its distinctive flavor, high nutritional value, and disease prevention [1,2,3]. Rapeseed oil can be divided into solvent extraction, cold pressing, and hot pressing, with hot-pressed rapeseed oil also known as fragrant rapeseed oil (FRO) [4]. Recently, FRO has become increasingly popular because of its unique flavor. FRO contains large amounts of unsaturated fatty acids, which are beneficial to health and can prevent cardiovascular and cerebrovascular diseases. In addition to a large amount of unsaturated fatty acids, FRO also contains bioactive substances such as tocopherols, sterols, and polyphenols, which are beneficial for improving nutritional value and oxidation stability [5,6,7,8]. Flavor is one of the most critical quality evaluation indicators for FRO. A previous report showed 137 volatile flavor compounds found in FRO, based on olfactory studies, including aldehydes, ketones, acids, esters, alcohols, phenols, pyrazines, furans, indole, pyridines, pyrrolines, thiazoles, thiophene, further S-contained compounds, nitriles, and alkenes [9].

Frying is one of the most common and popular food processing methods, which gives fried food an attractive flavor, color, and crisp taste, and is deeply loved by consumers [10]. However, as the frying process is conducted under high temperatures and intense processing conditions, various chemical reactions may occur during the frying process of edible oils, such as oxidation, hydrolysis, and polymerization reactions, so that the quality of oil decreases sharply during the frying process [11,12]. In recent years, many studies have found that most complex chemical reactions in the frying process lead to the degradation of oil, which affects the safety of fried food. In addition, the degradation of oil can easily produce many toxic and harmful compounds such as aldehydes, ketones, and trans fatty acids, which are harmful to human health [13,14,15,16]. A previous report showed that a large amount of (E,E)-2,4-decadienal produced after frying produces a pleasant deep-fried flavor, while the formation of hexanoic acid, heptanic acid, octanoic acid, and nonanoic acid is related to an unpleasant rancidity flavor [17]. Therefore, during the frying process, control of the deterioration of oil quality and flavor should be paid increasing attention.

Phenolic compounds are phytochemicals with important biological properties, which have been described to inhibit lipid oxidation by capturing peroxyradicals. A previous report demonstrated that erucic acid extract (isolated from rapeseed by-products) was more effective than a tocopherol mixture in inhibiting lipid oxidation in oil. Canolol is a phenolic compound formed by the decarboxylation of erucic acid during thermal induction, and approximately 56–83% of canolol is transferred to oil after pressing [18]. Recently, canolol has attracted considerable attention owing to its antioxidant, mutagenic, and anticancer properties [19,20,21]. Some studies have shown that an increase in polyphenol content can improve the thermal stability of FRO during frying and slow down the lipid oxidation rate of FRO during accelerated storage. The main materials used were phenolic mixtures. Moreover, the effect of polyphenols on FRO flavor retention has not been reported. Therefore, this study focused on the influence of canolol on the quality and flavor of FRO during frying.

In this study, the effects of high-canolol phenolic extracts (HCP) were evaluated by determining physicochemical indicators, fatty acid composition, and volatile flavor compounds, as well as the possible mechanism by which phenolic extracts affect the quality and flavor of FRO was preliminarily discussed. This study is expected to provide a reference for the quality and flavor of FRO in the frying process and provide a theoretical basis for the application of canolol.

## 2. Materials and Methods

### 2.1. Materials and Reagents

Fragrant rapeseed oil was purchased from Sichuan Hongfan Grain and Oil Co., Ltd. (Mianyang, China). French fries were purchased from Shanghai Changsheng Food Co., Ltd. (Shanghai, China). A mixed standard of 37 fatty acid methyl esters was purchased from Sigma-Aldrich (Sigma Chemical Co., St. Louis, MO, USA). Sinapic acid (98%), sinapine (97%), caffeic acid (99%), resveratrol (98%), canolol (97%), 2-methyl-3-heptanone (99%), hexanal (99%), (E,E)-2,4-decadienal (99%), hexanoic acid (98%), 2,5-dimethylpyrazine (99%), heptanenitrile (98%) standards were obtained from Aladdin Reagent Company (Shanghai, China). The rest of the reagents were purchased from Sinopharm Chemical Reagent Co., Ltd. (Shanghai, China).

### 2.2. Methods

#### 2.2.1. Extraction of Phenolic Compounds

The phenolic compounds were extracted using the modified procedures described by Servili et al. [22]. Methanol with a volume fraction of 70% was used as the extraction solvent, and the ratio of oil to solvent was 2 g/mL. After swirling at 2000 r/min for 6 min and centrifuged at 5000 r/min for 10 min, the hydro-methanolic phase was collected. The above operations were repeated 3 times. The hydro-methanolic extract was evaporated under a vacuum in a rotary evaporator (RE100-Pro, SCILOGEX, Rocky Hill, CT, USA) at 40 °C.

#### 2.2.2. Oil Samples Preparation

Three FRO models with different total phenol contents were prepared. The FRO was used as the control substance and named model FRO (MRO). The phenolic compounds extracted in 2.2.1 were added to the initial rapeseed oil to obtain model rapeseed oil with phenolic compounds content about 1.5 times and 2.0 times of MRO, respectively, named MRO+P and MRO++P.

#### 2.2.3. Simulated Frying Process

The simulated frying was carried out in a thermostatically temperature-controlled fryer (DF-40B electric fryer, Zhongshan Silede Electric Appliance Co., Ltd., Zhongshan, China) at 180 (±2) °C. The fryer was filled with 2 kg oil, and 12 batches of 100 g French fries were fried every day, each batch was fried for 5 min, then waited for the next frying cycle for 25 min. The whole frying process was carried out for 4 consecutive days and took 6 h every day [17]. No new oil was added during frying. Oil samples were taken at 0, 2, 4, 8, 12, 16, 20, and 24 h and immediately put into the refrigerator at 4 °C until analysis.

#### 2.2.4. Determination of Peroxide Value (PV), Acide Value (AV), p-Anisidine (p-AnV), Carbonyl Value (CV), Total Popar Compound Content (TPC)

The PV, AV, p-AnV, and TPC were determined according to the American Oil Chemists’ Society official method Cd 8b–90 [23], Da 14–48 [24], Cd 18–90 [25], and Cd 20–91 [26], respectively. The CV was determined according to the Japan Oil Chemists’ Society Official Method Tentative 13–2013 Carbonyl Value (Butanol Method) [27].

#### 2.2.5. Determination of Phenolic Compounds

A high pressure liquid chromatography (HPLC) system (Waters, Milford, MA, USA) combined with a binary pump(1525 model), autosampler(2707 model), diode array detector (2996 model), and data analysis software (Empower 3) was used. A binary solvent system consisting of methanol (solvent A) and water acidified with acetic acid (pH = 2.8) (solvent B) was used with a gradient elution on a Venusil MP C18(2) (4.6 mm × 250 mm, 5 μm) column at flow rate 1 mL/min as follows: 0 min, 20% A; 0~5 min, 20~30% A; 5~30 min, 30~50% A, 30~35 min, 50~20% A, and equilibrated at 20% A for 3 min. Sinapine, caffeic acid, resveratrol, and sinapic acid were measured at 320 nm, and canolol was detected at 280 nm. Injections of 20 μL were performed. External standard quantification was performed based on a series of five different standard concentrations. All analyses were duplicated.

#### 2.2.6. Determination of Fatty Acid Composition

The fatty acid composition of FRO was determined as previously described with slight modifications [28]. 50–55 mg FRO was taken into a 15 mL round-bottom centrifuge tube, 2 mL 0.5 mol/L NaOH-CH_3_OH solution was added, and the mixture was maintained in boiling water for 5 min. Then 2 mL BF_3_ solution was added, and the mixture was maintained in boiling water for 3 min. 2 mL saturated NaCl solution and 2 mL n-hexane were added after cooling. After blending, the n-hexane layer was absorbed and passed through a 0.22 μm organic filter membrane for detection.

GC analysis was performed by an Agilent 7890A GC system (Agilent, Santa Clara, CA, USA) equipped with a DB-WAX capillary column (30 m × 0.25 mm × 0.15 µm). Analyses were carried out at a split ratio of 50:1 with helium as the carrier gas at a flow rate of 0.8 mL/min. The oven temperature was initially held at 60 °C for 2 min, increased to 200 °C at a rate of 15 °C/min, and then increased to 230 °C at a rate of 3 °C/min, held at 230 °C for 25 min. All analyses were duplicated.

#### 2.2.7. Determination of Flavor Compounds

The assay of flavor compounds was performed as previously described [29] and optimized slightly. A 5 g FRO sample was added into a 15 mL headspace bottle sealed with a PTFE silicone padded lid. The aging extraction head was inserted and preheated at a constant temperature of 60 °C for 20 min. An activated SPME fiber (Supelco, Bellefonte, PA, USA) was inserted into the headspace vial. After adsorption for 50 min, the SPME fiber was inserted into the GC inlet, where thermal desorption at 250 °C was allowed to proceed for 8 min before data collection.

A GC-MS (QP-2010, Shimadzu, Shimane, Japan) equipped with a DB-5MS capillary column (60 m × 0.25 mm × 0.25 µm) was employed to analyze the samples. Analyses were carried out in splitless mode with helium as the carrier gas at a flow rate of 1.5 mL/min. The oven temperature was initially held at 50 °C for 2 min, increased to 180 °C at a rate of 3 °C/min, and then increased to 220 °C at a rate of 10 °C/min, held at 220 °C for 10 min. Ionization was performed in electron impact mode at 70 eV. Chromatograms were collected in full scan mode with a mass scan range of m/z 30–550.

Flavor compounds were qualitatively analyzed by standard compounds, mass spectra in the database (NIST18. LIB) [29], and retention index. The external standard method was used for the quantitative analysis of flavor compounds. Using a series of working standard solutions, the peak area corresponding to each standard in the mixture was determined at varying concentrations, and standard curves were constructed. The concentrations of the flavor compounds (µg/kg) in the oil samples were then determined from the standard curve using the corresponding peak areas, which were the average values from three measurements.

Based on molecular structure and functional groups, the flavor classes of FRO that can be detected in the paper are alcohols, aldehydes, acids, ketones, esters, hydrocarbons, heterocycles, and thiolaside degradation products.

#### 2.2.8. Evaluation Method of Main Flavor Compounds

*ROAV* method was used to evaluate the contribution of volatile compounds to the overall flavor of FRO at each oxidation stage, and the compound with the largest contribution to flavor was defined as *ROAV* = 100. The calculation formula is as follows:

$$ROAVi=100\times (\frac{Ci}{Cm}\times \frac{Tm}{Ti})$$

Type: *ROAV*i is the relative odor activity value of compound i; *C*i is the relative percentage of compound i; *T*i is the sensory threshold of compound i in vegetable oil found in the literature; *C*m and *T*m are the maxima of *C*i/*T*i among all the compounds in the sample. *ROAV* ≥ 1 indicates that the compound is the main volatile compound of the analyzed sample, and 0.1 ≤ *ROAV* < 1 indicates that the compound can modify the overall flavor [30].

#### 2.2.9. Sensory Evaluation

The sensory evaluation team consisted of 10 trained members (aged 20–30 years, 5 males and 5 females). The members were trained according to the China National Standard method (GB/T 16291). Sensory evaluation was performed in a sensory evaluation room at (21 ± 1) °C. Several aroma qualities were evaluated by descriptive testing, according to Zhang et al. [31]. Reference solutions for FRO were provided for each descriptor as follows: hexanal for grass, (E,E)-2,4-decadienal for deep-fried, hexanoic acid for rancid, 2,5-dimethylpyrazine for nutty, and heptanenitrile for pungent. The intensities of the selected odor attributes (grass, deep-fried, rancid, nutty, pungent) were noted by trained sensory members on a linear scale from 0 (not perceptible) to 10 (strongly perceptible) in steps of 1.0. Each member assessed each sample three times.

#### 2.2.10. Statistical Analysis

Data were expressed as mean ± standard deviation (SD) from triplicate determinations. The analysis of variance (ANOVA) was performed with SPSS 22.0 (IBM, Armonk, NY, USA), and the Tukey test was used to determine significant differences between means (*p* < 0.05).

## 3. Results and Discussion

### 3.1. Characterization of Oil Samples

The results of HCP in FRO are listed in Table 1. Five phenolic compounds (sinapine, caffeic acid, resveratrol, sinapic acid, and canolol) were identified. Canolol was the main phenolic compound in FRO, accounting for approximately 91% of the total phenol content. The result is very close to that reported by Kraljic et al., which states that Canolol content is 85% of the total phenol content [32]. Therefore, this study mainly focused on the effects of canolol. The total phenol content of model rapeseed MRO, MRO+P, and MRO++P were 181.48 ± 3.79 (Canolol: 165.17 ± 2.68), 276.34 ± 4.50 (Canolol: 251.47 ± 3.97), and 367.77 ± 5.22 mg/kg (Canolol: 334.67 ± 4.87), respectively.

### 3.2. Effects of HCP on Physicochemical Indicators of Frying Oil

**Figure 1:**
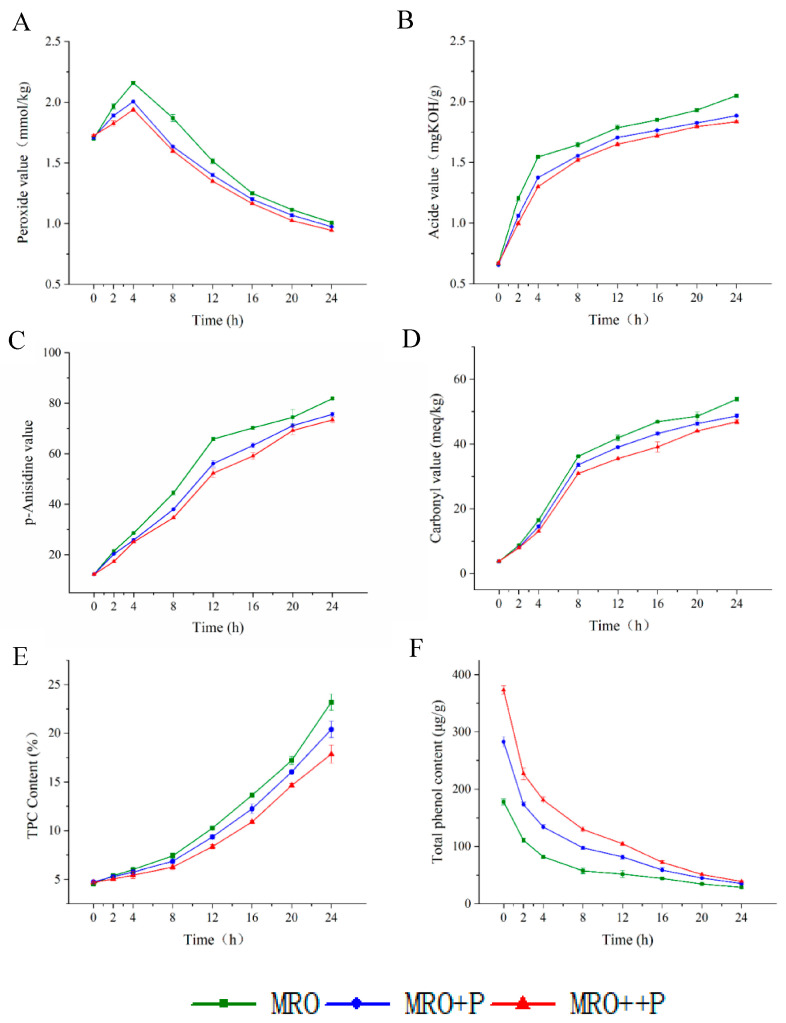
Changes of physicochemical properties of oil during frying process. (**A**) Peroxide value, (**B**) Acid value, (**C**) p-Anisidine value, (**D**) Carbonyl value, (**E**) Total polar compound content, (**F**) Total phenol content. Data are the mean of two independent experiments analyzed twice.

Oil quality is very important for frying performance. Usually, PV, AV, p-AnV, CV, and TPC are used as physicochemical indicators to evaluate frying oil quality [10]. Figure 1A shows the changes in the PV of FRO at different frying times. The PV first increased and then decreased with the extension of frying time. Gao et al. reported a similar result for tea polyphenols on the quality of rapeseed oil during the frying process [33]. At 4 h, the PV of MRO reached a maximum value of 2.16 mmol/kg, which was higher than that of MRO+P (2.01 mmol/kg) and MRO++P (1.94 mmol/kg), which indicated that HCP could inhibit oil oxidation during frying. The AV, p-AnV, CV, and TPC showed similar increasing trends. The polyphenols performed well in inhibiting the increase of TPC, AV, and p-AnV [26]. Figure 1B shows the changes in the AV of FRO at different frying times. The AV of MRO reached a maximum value of 2.05 mgKOH/g at 24 h, which was higher than that of MRO+P (1.89 mgKOH/g) and MRO++P (1.84 mgKOH/g). Figure 1C shows the changes in the p-AnV of FRO at different frying times. It can be clearly seen that the p-AnV of MRO reached a maximum value of 81.84 at 24 h, which was higher than that of MRO+P (75.62) and MRO++P (73.41). Figure 1D shows the changes in the CV of FRO at different frying times. The CV of the MRO reached a maximum value of 53.87 meq/kg at 24 h, which was higher than that of MRO+P (48.68 meq/kg) and MRO++P (46.91 meq/kg). Figure 1E shows the changes in the TPC of FRO at different frying times. The TPC of MRO reached a maximum value of 23.20% at 24 h, which was higher than that of MRO+P (20.40%) and MRO++P (17.85%). Figure 1F shows the changes in total phenol content in the three oil samples during the frying process. The trends of the three oil samples were generally consistent, showing a decreasing trend, and the total phenol content of the three oil samples was close to each other at 24 h, the results are consistent with the trend in Kasprzak et al. [34]. The loss of phenolic compounds at the end of the frying process can be attributed to the oxidation or hydrolysis associated with thermal processing. From the above analysis, it can be seen that HCP can effectively inhibit the oxidation and secondary oxidation of FRO, prolonging the optimal stage and showing good frying properties, which is beneficial for the improvement of the deep-fried flavor.

### 3.3. Effects of HCP on Fatty Acids Composition

The fatty acids composition is an important indicator for evaluating the quality of frying oil. The results of the percentage composition of fatty acids for MRO, MRO+P, and MRO++P after frying at 180 °C for 0, 8, 16, and 24 h are shown in Table 2. Compared with the unsaturated fatty acids content of fresh oil, the unsaturated fatty acids content of each sample significantly decreased (*p* < 0.05). The unsaturated fatty acids content of MRO+P and MRO++P was 81.97% and 82.59%, respectively. This was significantly higher than that of MRO (80.85%) (*p* < 0.05), indicating that HCP can inhibit the degradation of unsaturated fatty acids during frying, thus effectively protecting the quality of FRO, Aniolowska et al. reported a similar result on the changes of fatty acids composition during frying process [35]. In general, the quantity and type of unsaturated fatty acids may have a significant effect on the nutritional value and quality of fatty foods. It has been reported that unsaturated fatty acids in daily diets have various physiological functions such as lowering blood lipids, anti-thrombosis, and immune regulation, which are beneficial to human health [36].

### 3.4. Effects of HCP on Flavor Compounds

**Figure 2:**
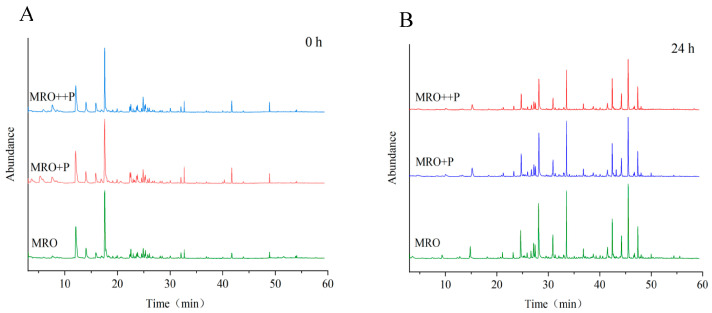
Chromatograms of the flavor compounds extracted by SPME from the oil samples frying for 0 h (**A**) and 24 h (**B**).

Figure 2A,B show the total ion flow diagrams of flavor compounds in MRO, MRO+P, and MRO++P frying for 0 and 24 h, respectively. As shown in Figure 2A, the contents of the main flavor compounds in the three initial oil samples were not significantly different. However, Figure 2B shows that after frying for 24 h, the total peak areas of MRO+P and MRO++P were significantly lower than that of MRO, indicating that HCP could inhibit the generation of volatile flavor compounds.

**Figure 3:**
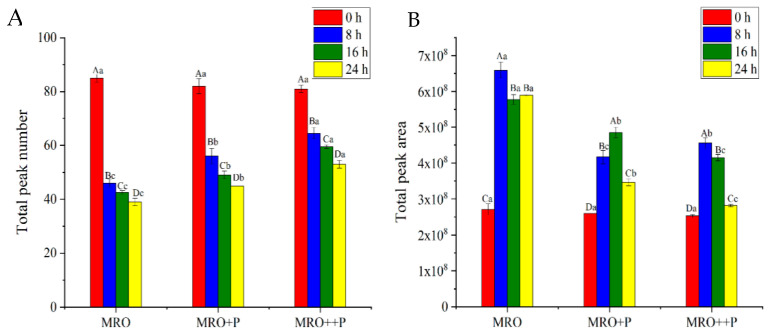
Changes of the total peak number (**A**) and area (**B**) of oil during frying process. Note: Different letters (A–D) indicate significant differences between times for the same oil samples (*p* < 0.05); Different letters (a–c) indicate significant differences between oil samples for the same step of frying process (*p* < 0.05). Data are the mean of three independent experiments analyzed twice.

Figure 3A,B show the changes in the total peak number and total peak area of flavor compounds, respectively, in MRO, MRO+P, and MRO++P during frying. After frying for 24 h, the total peak number of the three oil samples decreased significantly (*p* < 0.05), and the decreased amplitude was the largest during 0–8 h. Meanwhile, the total peak number of MRO+P and MRO++P decreased significantly compared to that of MRO, indicating that HCP can significantly reduce the total peak number during the frying process. The total peak area of the three oil samples showed a rapidly increasing trend within 0–8 h, which was mainly caused by the oxidation degradation of the oxidation products of FRO during heating to generate small-molecule aldehydes, alcohols, and short-chain fatty acids. Meanwhile, the total peak area of MRO+P and MRO++P was significantly lower than that of MRO (*p* < 0.05), indicating that HCP could significantly inhibit the oxidation of FRO during frying, thus reducing the formation of off-flavors.

Table 3 shows the types and concentrations of flavor compounds during the frying process. There were 13 types of alcohol compounds in MRO and MRO++P before frying, mainly hexanol, heptanol, 1-octene-3-ol, and octanol, which were related to the green flavor of FRO. The relative content of alcohol compounds showed a decreasing trend during frying, but no significant difference was found among the three oil samples. The results showed that HCP had no significant effect on the change in the alcohol content during frying. Aldehydes are mainly simple saturated aldehydes when they are not fried, including hexanal, octanal, and nonanal. These aldehydes can provide some grass flavor at low concentrations but produce a rancid flavor at high concentrations [37]. The relative contents of these aldehydes increased at the end of frying, and with the extension of frying time, the relative contents of (E)-2-heptenal, (E,E)-2,4-heptadienal, (E)-2-octenal, (E)-2-decenal, and (E,E)-2,4-decadienal showed a trend of first increasing and then decreasing, and these enaldehydes and dienaldehydes were related to a pleasant deep-fried flavor. After frying for 24 h, the relative content of aldehydes in MRO increased by 64.4%, whereas that in MRO++P only increased by 56.9%, indicating that HCP can inhibit the generation of aldehydes (*p* < 0.05), which may be related to the inhibition of unsaturated fatty acid degradation by HCP. The relative content of acid compounds, mainly small molecule acids such as hexanoic, heptanoic, and octanoic acids, increases with the extension of frying time. These acidic compounds are related to the rancid flavor. The relative contents of caproic, heptanic, and octanic acids increased from 3.08%, 3.10%, and 3.11% to 6.36%, 5.87%, and 5.29%, respectively, after frying for 24 h, indicating that HCP can inhibit the generation of acid compounds. With the extension of frying time, the relative ketone content decreased rapidly, and the number of ketones in MRO decreased from 9 to 1 after frying for 8 h, while the number of ketones in MRO+P and MRO++P decreased from 9 and 10 to 3 and 6, respectively, after frying for 8 h. The relative content of MRO, MRO+P, and MRO++P decreased from 17.8%, 16.9%, and 16.6% to 4.28%, 6.38%, and 7.98%, respectively, indicating that HCP significantly slowed the loss of ketones (*p* < 0.05). The ester compounds were significantly reduced after frying, and no ester compounds were detected in any of the three oil samples 8 h after frying, indicating that phenolic compounds had no significant effect on the change in ester compounds during frying. The relative hydrocarbon content decreased during the frying process, but no significant difference was found among the three oil samples, indicating that HCP had no significant effect on the change in hydrocarbons during frying. Heterocycles are mainly related to the nut aroma of FRO. The relative content of heterocycles decreased rapidly in the first 8 h of frying and decreased from 25.84%, 23.91%, and 24.53% to 4.73%, 9.94%, and 12.43%, respectively, after 24 h of frying. The results showed that HCP significantly slowed the loss of heterocycles (*p* < 0.05). Thiolaside degradation products decreased rapidly after frying, but no significant difference was found among the three oil samples, indicating that HCP had no significant effect on the change in thiolaside degradation products during frying. The results showed that HCP significantly inhibited aldehydes, acids, ketones, and heterocyclic compounds.

### 3.5. Effects of HCP on Characteristic Flavor Compounds

In this study, 67 sensory thresholds of volatile flavor compounds were retrieved. It is generally believed that the group with relative odor activity value (ROAV) ≥ 1 was divided into the main flavor compounds of the tested samples, and the larger the ROAV, the greater the contribution to the overall flavor of the samples. The compound with 0.1 ≤ ROAV < 1 played an important role in the modification of the overall flavor of the tested samples. Table 4 shows the effects of HCP on the concentrations of the characteristic flavor compounds during the frying process.

Table 4 shows that the main flavor compounds in FRO frying for 0 h included hexanoic acid, octanoic acid, nonanoic acid, (E)-2-heptenal, heptanol, 1-octene-3-ol, dimethyl trisulfide, 2-n-pentylfuran, (E)-2-octenal, octanol, nonal, and (E)-2-decenal. After frying, the main flavor compounds of FRO included hexanoic acid, octanoic acid, nonanoic acid, hexanal, (E)-2-heptenal, n-heptanol, 1-octene-3-ol, 2-n-pentylfuran, (E)-2-octenal, (E,E)-2,4-heptadienal, nonal, (E,E)-2,4-nonanodienal, (E)-2-decenal, and (E,E)-2,4-decdienal. Zhang et al. summarized that 137 odorants were found in rapeseed oil and the results above are consistent with those main odorants [38]. With the extension of frying time, volatile flavor compounds showed an increasing trend, especially saturated aldehydes and acid compounds such as hexanoic acid, octanoic acid, nonanoic acid, hexanal, and nonanal. The octanoic acid content increased the fastest in 0–8 h, and the octanoic acid contents of MRO, MRO+P, and MRO++P increased by 562.50%, 480.00%, and 318.75%, respectively, indicating that HCP significantly inhibited the generation of octanoic acid (*p* < 0.05). (E)-2-heptenal, (E)-2-octenal, (E,E)-2,4-heptenal, (E)-2-decenal, (E,E)-2,4-decadienal, and other aldehydes showed a trend of first increasing and then decreasing with the extension of frying time, and (E,E)-2,4-decadienal reached its maximum value after frying for 16 h. From 16 to 24 h, the (E,E)-2,4-decadiene content of MRO, MRO+P, and MRO++P decreased by 14.70%, 13.29%, and 11.72%, respectively, indicating that HCP significantly inhibited the degradation of (E,E)-2,4-decadienal (*p* < 0.05), and similar results were found in the study of Xu et al. [26]. This may be due to the fact that these unsaturated aldehydes are further oxidized to saturated aldehydes with frying, and saturated aldehydes are further oxidized to the corresponding acids. It has previously been reported that the generation of aldehydes is related to the degradation of unsaturated fatty acids during heating. Nonanal is generated by oleic acid degradation, whereas hexanal and (E,E)-2,4-decadienal are generated by linoleic acid degradation. (E,E)-2,4-heptadienal is produced by the degradation of linolenic acid [39]. Octanoic acid and nonanoic acid are generated from the oxidative degradation of oleic acid and are related to the rancid flavor generated in the later frying period. The increase in these saturated aldehydes and acids causes the off-flavor of FRO. In addition, (E,E)-2,4-decadienal can generate a pleasant deep-fried flavor. HCP significantly inhibited the degradation of (E,E)-2,4-decadienal and slowed down the increase in acid compounds (*p* < 0.05), which might be related to the inhibition of unsaturated fatty acid degradation by HCP during frying [26].

### 3.6. Effects of HCP on Sensory Evaluation

**Figure 4:**
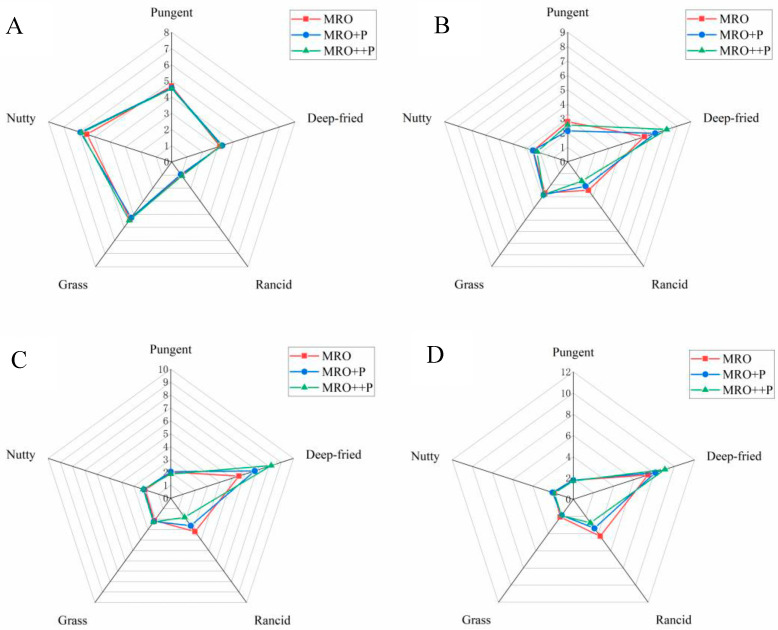
Fragrant rapeseed oil flavor sensory evaluation radar charts. Note: (**A**–**D**) respectively represent the sensory analysis results of the three oil samples after frying for 0, 8, 16 and 24 h.

The sensory analysis results of the three oil samples after frying for different times (0, 8, 16, and 24 h) are shown in Figure 4A–D. The pungent, nutty, and grass flavor scores of the three oil samples were higher before frying. With the frying process, the scores for the above three flavors decreased rapidly. The scores for deep-fried and rancid flavors showed an increasing trend. The results showed that HCP had no significant effect on the pungent, grass, and nutty flavors, but had a positive effect on the deep-fried flavor of FRO, which could slow down the reduction of the deep-fried flavor and inhibit the formation of the rancid flavor, which was consistent with the results of 3.4 and 3.5.

## 4. Conclusions

The results showed that during the frying process, HCP can significantly inhibit the degradation of some flavor compounds, such as (E,E)-2,4-decadienal, which is associated with a pleasant deep-fried flavor and can inhibit the generation of some off-flavor compounds (hexanoic acid, nonanoic acid, etc.), which are associated with the rancid off-flavor. HCP significantly inhibited the deterioration of the physicochemical properties of FRO and the degradation of unsaturated fatty acids, which could prolong the usability of FRO. Therefore, the results indicate that HCP has potential application value in the quality protection and flavor improvement of FRO during frying.

## Figures and Tables

**Table 1:** The phenolic extracts composition (mg/kg) of the three initial oil samples.

	MRO	MRO+P	MRO++P
Sinapine	5.81 ± 0.52 ^c^	7.52 ± 0.43 ^b^	11.77 ± 0.56 ^a^
Caffeic acid	3.63 ± 0.41 ^c^	4.70 ± 0.32 ^b^	7.36 ± 0.43 ^a^
Resveratrol	3.27 ± 0.35 ^c^	4.23 ± 0.41 ^b^	6.62 ± 0.42 ^a^
Sinapic acid	14.52 ± 0.87 ^c^	18.79 ± 1.12 ^b^	29.42 ± 0.97 ^a^
Canolol	154.32 ± 3.54 ^c^	234.89 ± 4.01 ^b^	312.61 ± 4.86 ^a^
Total phenol content (HPLC)	181.48 ± 3.79 ^c^	276.34 ± 4.50 ^b^	367.77 ± 5.22 ^a^

Note: Values are the average of three replicates of analysis. Values followed by different letters are significantly different (*p* < 0.05).

**Table 2:** The fatty acid composition of oil during frying.

Fatty Acids		Fatty Acids Composition (%)								
		C16:0	C18:0	C18:1	C18:2	C18:3	C20:1	C22:1	SFA	MUFA
0 h	MRO	6.82 ± 0.02 ^Da^	2.87 ± 0.02 ^Ca^	49.95 ± 0.22 ^Ca^	30.5 ± 0.19 ^Aa^	8.15 ± 0.04 ^Ab^	0.92 ± 0 ^Bb^	0.8 ± 0.02 ^Aa^	9.69 ± 0.03 ^Da^	90.31 ± 0.03 ^Aa^
	MRO+P	6.85 ± 0.02 ^Da^	2.67 ± 0.03 ^Db^	50.44 ± 0.23 ^Ca^	30.21 ± 0.25 ^Aa^	8.25 ± 0.03 ^Aa^	0.91 ± 0.01 ^ABb^	0.68 ± 0.02 ^Bb^	9.51 ± 0.04 ^Db^	90.49 ± 0.04 ^Aa^
	MRO++P	6.78 ± 0.08 ^Da^	2.65 ± 0.01 ^Db^	50.47 ± 0.2 ^Da^	30.25 ± 0.22 ^Aa^	8.31 ± 0.01 ^Aa^	0.95 ± 0.01 ^Aa^	0.6 ± 0.02 ^Ac^	9.43 ± 0.08 ^Db^	90.57 ± 0.08 ^Aa^
8 h	MRO	8.77 ± 0.06 ^Ca^	2.87 ± 0.02 ^Ca^	53.26 ± 0.12 ^Aa^	26.2 ± 0.06 ^Bc^	7.22 ± 0.06 ^Ba^	0.93 ± 0.01 ^Ba^	0.74 ± 0.01 ^Ba^	11.65 ± 0.04 ^Cab^	88.35 ± 0.04 ^Bab^
	MRO+P	8.79 ± 0.04 ^Ca^	2.94 ± 0.03 ^Cb^	52.29 ± 0.11 ^Bb^	27.56 ± 0.06 ^Bb^	6.98 ± 0.1 ^Bb^	0.91 ± 0.02 ^ABa^	0.53 ± 0 ^Cc^	11.73 ± 0.07 ^Ca^	88.27 ± 0.07 ^Bb^
	MRO++P	8.55 ± 0.12 ^Cb^	2.93 ± 0.03 ^Cb^	51.79 ± 0.15 ^Cc^	28.2 ± 0.08 ^Ba^	7.06 ± 0.04 ^Bab^	0.92 ± 0.01 ^Ba^	0.55 ± 0.01 ^Bb^	11.48 ± 0.14 ^Cb^	88.52 ± 0.14 ^Ba^
16 h	MRO	13.74 ± 0.15 ^Ba^	3.14 ± 0.04 ^Ba^	51.66 ± 0.33 ^Bc^	23.67 ± 0.22 ^Cc^	6.13 ± 0.06 ^Ca^	0.96 ± 0.01 ^Aa^	0.7 ± 0.01 ^Cb^	16.88 ± 0.18 ^Ba^	83.12 ± 0.18 ^Cc^
	MRO+P	12.31 ± 0.07 ^Bb^	3.15 ± 0.01 ^Ba^	52.96 ± 0.2 ^Ab^	24.21 ± 0.22 ^Cb^	5.61 ± 0.04 ^Cb^	0.96 ± 0.04 ^Aa^	0.8 ± 0.04 ^Aa^	15.47 ± 0.06 ^Bb^	84.53 ± 0.06 ^Cb^
	MRO++P	11.26 ± 0.04 ^Bc^	3.10 ± 0.04 ^Ba^	53.75 ± 0.14 ^Aa^	25.06 ± 0.06 ^Ca^	5.41 ± 0.04 ^Cc^	0.9 ± 0.01 ^Cb^	0.52 ± 0.01 ^Cc^	14.37 ± 0.07 ^Bc^	85.63 ± 0.07 ^Ca^
24 h	MRO	15.87 ± 0.14 ^Aa^	3.28 ± 0.02 ^Aa^	51.72 ± 0.49 ^Bb^	22.46 ± 0.36 ^Db^	5.08 ± 0.03 ^Db^	0.95 ± 0.01 ^Aa^	0.64 ± 0.02 ^Db^	19.15 ± 0.17 ^Aa^	80.85 ± 0.17 ^Dc^
	MRO+P	14.52 ± 0.31 ^Ab^	3.51 ± 0.03 ^Ab^	51.97 ± 0.44 ^Bb^	22.78 ± 0.16 ^Dab^	5.56 ± 0.13 ^Ca^	0.89 ± 0.02 ^Bb^	0.77 ± 0.02 ^Aa^	18.03 ± 0.27 ^Ab^	81.97 ± 0.27 ^Db^
	MRO++P	13.83 ± 0.1 ^Ac^	3.58 ± 0.05 ^Ab^	53.28 ± 0.07 ^Ba^	23.34 ± 0.1 ^Da^	4.56 ± 0.04 ^Dc^	0.88 ± 0 ^Db^	0.54 ± 0 ^BCc^	17.41 ± 0.12 ^Ac^	82.59 ± 0.12 ^Da^

Note: Average value of all data points ± standard deviation; Different letters (A–D) indicate significant differences between times for the same oil samples (*p* < 0.05); Different letters (a–c) indicate significant differences between oil samples for the same step of frying process (*p* < 0.05); C16:0, palmiticacid; C18:0, estearic acid; C18:1, oleic acid; C18:2, linoleic acid; C18:3, α-linolenic acid; C20:1, arachidic acid; C22:1, erucic acid; SFA, Saturated fatty acids; MUFA, monounsaturated fatty acids. Data are the mean of three independent experiments analysed twice.

**Table 3:** Effects of HCP on the kinds and concentrations of flavor compounds during frying.

Compounds	The Number and Relative Content of Flavor Compounds																							
	0 h						8 h						16 h						24 h					
	MRO		MRO+P		MRO++P		MRO		MRO+P		MRO++P		MRO		MRO+P		MRO++P		MRO		MRO+P		MRO++P	
	A	B	A	B	A	B	A	B	A	B	A	B	A	B	A	B	A	B	A	B	A	B	A	B
alcohols	13	7.38	13	7.51	13	7.45	9	5.96	10	5.89	11	5.79	8	5.54	8	5.61	10	5.58	6	4.01	6	4.13	9	4.11
aldehydes	13	17.4	12	16.9	12	17.4	19	74.48	20	70.11	18	69.08	18	77.95	19	74.38	20	72.04	21	82.28	18	76.84	21	74.01
acids	7	3.08	7	3.1	8	3.11	3	4.67	5	4.41	6	4.03	4	5.49	3	5.23	4	4.93	4	6.36	4	5.87	5	5.29
ketones	9	17.8	9	16.9	10	16.6	1	4.28	3	6.38	6	7.98	1	3.11	2	3.27	5	4.02	1	1.92	1	1.99	3	2.43
esters	5	0.08	4	0.14	5	0.15	ND	ND	1	0.11	1	0.12	ND	ND	ND	ND	ND	ND	ND	ND	ND	ND	ND	ND
hydrocarbons	9	2.95	10	3.01	9	3.21	5	1.39	7	1.37	7	1.41	4	0.87	6	0.88	7	0.91	2	0.81	5	0.79	6	0.83
heterocycles	15	25.84	15	23.91	12	24.53	7	8.07	9	10.04	9	9.66	8	6.48	10	9.96	9	11.49	6	4.73	11	9.94	9	12.43
thiolaside degradation products	15	25.47	16	28.53	13	27.55	3	2.25	4	2.59	6	2.53	1	1.56	2	1.67	5	1.63	1	1.39	2	1.44	3	1.4

Note: A represents the number of compounds; B (%) represents the relative content of compounds; ND, not detected under this analysis condition. Data are the mean of three independent experiments analysed twice.

**Table 4:** Effects of HCP on concentrations of characteristic flavor compounds during frying.

Compounds						Concentration (μg/g)											
	Odor Description	ROAV				0 h			8 h			16 h			24 h		
		0 h	8 h	16 h	24 h	MRO	MRO+P	MRO++P	MRO	MRO+P	MRO++P	MRO	MRO+P	MRO++P	MRO	MRO+P	MRO++P
Hexanoic acid	sweaty	1.59	0.69	0.59	0.63	0.08 ± 0.03 ^Da^	0.08 ± 0.02 ^Da^	0.08 ± 0.02 ^Da^	1.22 ± 0.04 ^Ca^	1.03 ± 0.05 ^Cb^	0.79 ± 0.03 ^Cc^	3.08 ± 0.09 ^Ba^	2.42 ± 0.06 ^Bb^	2.05 ± 0.07 ^Bc^	5.08 ± 0.09 ^Aa^	3.82 ± 0.06 ^Ab^	2.45 ± 0.07 ^Ac^
Octanoic acid	sweat, cheese, rancid	2.51	2.03	2.12	1.89	0.16 ± 0.04 ^Da^	0.15 ± 0.03 ^Da^	0.16 ± 0.04 ^Da^	1.06 ± 0.05 ^Ca^	0.87 ± 0.06 ^Cb^	0.67 ± 0.03 ^Cc^	1.42 ± 0.04 ^Ba^	1.24 ± 0.06 ^Bb^	0.95 ± 0.05 ^Bc^	2.25 ± 0.03 ^Aa^	1.92 ± 0.05 ^Ab^	1.21 ± 0.07 ^Ac^
Nonanoic acid	green, fat, rancid	1.44	1.35	1.09	1.11	0.25 ± 0.06 ^Da^	0.25 ± 0.04 ^Da^	0.26 ± 0.05 ^Da^	0.98 ± 0.04 ^Ca^	0.75 ± 0.03 ^Cb^	0.62 ± 0.04 ^Cc^	1.19 ± 0.05 ^Ba^	1.02 ± 0.04 ^Bb^	0.75 ± 0.04 ^Bc^	2.04 ± 0.05 ^Aa^	1.45 ± 0.03 ^Ab^	0.98 ± 0.04 ^Ac^
Hexanal	fat, citrus, rancid	1.01	0.90	1.62	1.70	0.78 ± 0.03 ^Da^	0.82 ± 0.05 ^Da^	0.86 ± 0.05 ^Da^	6.60 ± 0.05 ^Ca^	4.9 ± 0.05 ^Cb^	2.95 ± 0.06 ^Cc^	9.09 ± 0.04 ^Ba^	7.62 ± 0.06 ^Bb^	5.87 ± 0.07 ^Bc^	11.18 ± 0.33 ^Aa^	9.29 ± 0.13 ^Ab^	6.42 ± 0.06 ^Ac^
(E)-2-Heptenal	soap, fat, almond	7.33	67.16	72.37	73.49	0.32 ± 0.04 ^Da^	0.35 ± 0.05 ^Da^	0.28 ± 0.02 ^Da^	9.78 ± 0.05 ^Ca^	7.32 ± 0.15 ^Cb^	5.81 ± 0.12 ^Cc^	13.23 ± 0.24 ^Aa^	11.90 ± 0.08 ^Ab^	10.12 ± 0.1 ^Ac^	10.8 ± 0.1 ^Ba^	9.54 ± 0.13 ^Bb^	8.08 ± 0.05 ^Bc^
1-Heptanol	chemical, green	ND	1.30	3.25	3.31	ND	ND	ND	0.65 ± 0.03 ^Ba^	0.49 ± 0.05 ^Bb^	0.30 ± 0.02 ^Bc^	0.81 ± 0.05 ^Aa^	0.63 ± 0.02 ^Ab^	0.32 ± 0.02 ^Bc^	0.37 ± 0.03 ^Cb^	0.41 ± 0.02 ^Cb^	1.04 ± 0.02 ^Aa^
1-Octen-3-ol	mushroom	ND	100	100	100	ND	ND	ND	2.06 ± 0.06 ^Ba^	1.57 ± 0.06 ^Cb^	1.37 ± 0.05 ^Cc^	2.05 ± 0.04 ^Ba^	1.85 ± 0.02 ^Bb^	1.71 ± 0.03 ^Bc^	3.07 ± 0.1 ^Aa^	2.84 ± 0.12 ^Aa^	2.54 ± 0.08 ^Ab^
Dimethyl trisulfide	sulfur, fish, cabbage	5.72	ND	ND	ND	0.17 ± 0 ^Aa^	0.18 ± 0 ^Aa^	0.17 ± 0.01 ^Aa^	ND	ND	ND	ND	ND	ND	ND	ND	ND
2-Pentylfuran	green bean, butter	1.32	1.79	2.26	1.95	0.99 ± 0.03 ^Ca^	0.96 ± 0.02 ^Da^	1.01 ± 0.03 ^Da^	3.19 ± 0.04 ^Ba^	2.75 ± 0.04 ^Cb^	1.83 ± 0.06 ^Cc^	3.61 ± 0.07 ^Aa^	3.18 ± 0.13 ^Bb^	2.28 ± 0.07 ^Bc^	3.73 ± 0.13 ^Aa^	3.59 ± 0.04 ^Ab^	2.69 ± 0.04 ^Ac^
(E)-2-Octenal	green, nut, fat	8.81	57.32	59.24	60.78	0.36 ± 0.02 ^Da^	0.34 ± 0.03 ^Da^	0.31 ± 0.02 ^Da^	5.8 ± 0.19 ^Ca^	5.17 ± 0.1 ^Cb^	4.71 ± 0.09 ^Cc^	11.91 ± 0.17 ^Aa^	10.41 ± 0.29 ^Ab^	9.07 ± 0.04 ^Ac^	7.46 ± 0.06 ^Ba^	6.41 ± 0.09 ^Bb^	6.29 ± 0.08 ^Bc^
(E,E)-2,4-Heptadienal	nut, fat	2.58	2.93	6.91	5.91	1.01 ± 0.03 ^Da^	0.97 ± 0.06 ^Da^	1.06 ± 0.05 ^Ca^	28.58 ± 0.38 ^Aa^	27.91 ± 0.23 ^Ab^	25.95 ± 0.71 ^Ab^	25.79 ± 0.24 ^Ca^	24.32 ± 0.2 ^Cb^	22.32 ± 0.81 ^Bc^	26.84 ± 0.13 ^Ba^	24.93 ± 0.06 ^Bb^	22.29 ± 0.88 ^Bc^
1-Octanol	chemical, metal, burnt	3.04	1.20	1.52	1.97	0.43 ± 0.01 ^Da^	0.44 ± 0.03 ^Da^	0.48 ± 0.04 ^Da^	1.39 ± 0.03 ^Ca^	0.81 ± 0.03 ^Cb^	0.64 ± 0.04 ^Cc^	1.57 ± 0.05 ^Ba^	1.22 ± 0.02 ^Bb^	1.06 ± 0.03 ^Bc^	1.76 ± 0.07 ^Aa^	1.58 ± 0.02 ^Ab^	1.44 ± 0.02 ^Ac^
Nonanal	fat, citrus, green	1.31	2.47	2.95	3.20	1.45 ± 0.01 ^Da^	1.35 ± 0.09 ^Ca^	1.32 ± 0.04 ^Da^	18.93 ± 0.39 ^Ca^	12.94 ± 0.07 ^Bb^	8.33 ± 0.15 ^Cc^	22.52 ± 0.44 ^Ba^	13.83 ± 0.55 ^Bb^	12.71 ± 0.13 ^Bc^	29.86 ± 0.31 ^Aa^	24.23 ± 0.82 ^Ab^	19.43 ± 0.26 ^Ac^
(E,E)-2,4-Nonadienal	fat, wax, green	ND	13.52	12.52	12.67	ND	ND	ND	0.86 ± 0.02 ^Ca^	0.72 ± 0.01 ^Cb^	0.62 ± 0.02 ^Cc^	1.59 ± 0.04 ^Aa^	1.49 ± 0.04 ^Ab^	1.07 ± 0.04 ^Ac^	1.41 ± 0.04 ^Ba^	1.32 ± 0.05 ^Ba^	0.97 ± 0.02 ^Bb^
(E)-2-Decenal	tallow	2.67	4.87	4.39	4.11	0.73 ± 0.01 ^Da^	0.7 ± 0.04 ^Da^	0.71 ± 0.03 ^Da^	10.65 ± 0.11 ^Ca^	8.88 ± 0.09 ^Cb^	7.34 ± 0.16 ^Cc^	14.59 ± 0.31 ^Aa^	13.26 ± 0.58 ^Ab^	11.44 ± 0.12 ^Ac^	13.51 ± 0.33 ^Ba^	11.55 ± 0.31 ^Bb^	10.19 ± 0.08 ^Bc^
(E,E)-2,4-Decadienal	deep-fried, wax, fat	1.71	2.69	1.94	1.58	3.71 ± 0.07 ^Da^	3.71 ± 0.11 ^Da^	3.69 ± 0.09 ^Ca^	22.16 ± 0.69 ^Bc^	24.42 ± 0.3 ^Cb^	25.9 ± 0.12 ^Ca^	23.67 ± 0.18 ^Ac^	28.98 ± 0.34 ^Bb^	31.56 ± 0.3 ^Ba^	20.19 ± 0.04 ^Bc^	25.13 ± 0.76 ^Ab^	27.86 ± 0.68 ^Aa^

Note: Average value of all data points ± standard deviation; Different letters (A–D) indicate significant differences between times for the same oil samples (*p* < 0.05); Different letters (a–c) indicate significant differences between oil samples for the same step of frying process (*p* < 0.05); ND, not detected under this analysis condition. Data are the mean of three independent experiments analysed twice.

## Data Availability

All data included in this study are available upon request by contact with the corresponding author.
